# Silencing HE4 alleviates the renal fibrosis in lupus nephritis mice by regulating the C3/MMPs/prss axis

**DOI:** 10.1007/s00210-023-02883-x

**Published:** 2023-12-29

**Authors:** Yixia Li, Xiaorong Zhong, Feng Yang

**Affiliations:** Department of Nephrology, Affiliated Fuzhou First Hospital of Fujian Medical University, No.190, Dadao Road, Taijiang District, Fuzhou City, Fujian 350004 China

**Keywords:** MRL/LPR mice, HE4, Adenovirus, C3, MMPs

## Abstract

To explore the regulatory effect of human epididymis protein 4 (HE4) on renal fibrosis in mice with lupus nephritis (LN) and the underlying mechanism. Ten-week old MRL/LPR mice were injected with HE4 shRNA adenovirus vector through the renal pelvis for 5 days. Renal tissues were extracted for HE and Masson staining to evaluate pathological changes and fibrosis in lupus nephritis mice. The level of urine protein was measured using a biochemical analyzer, while the expression level of HE4 and p-NF-κB p65 in renal tissues was visualized using an immunofluorescence assay. The level of β2-microglobulin (β2-MG), neutrophil gelatinase-associated lipocalin (NGAL), and kidney injury molecule 1 (Kim-1) was determined by the immunohistochemical assay. Western blotting was used to determine the levels of C3, HE4, matrix metalloproteinase-2 (MMP2), MMP9, p-p65, prss23, and prss35 in renal tissues. Compared to wild-type C57BL/6 mice, MRL/LPR mice showed a marked increase in the number of glomeruli, hyperplasic basement membrane, severe infiltration of inflammatory cells in renal tubules and glomeruli, obvious necrosis in glomeruli, elevated fibrosis levels, and increased levels of urine protein, β2-MG, NGAL, Kim-1, C3, HE4, MMP2, MMP9, and p-p65; and decreased levels of prss23 and prss35 were observed in MRL/LPR mice. After the administration of the HE4 shRNA adenovirus vector, the repaired structure of renal tubules and glomeruli improved infiltration of inflammatory cells, reduced collagen fiber and urine protein, suppressed levels of C3, HE4, MMP2, MMP9, and p-P65, and facilitated the expression of prss23 and prss35 which were observed. Silencing HE4 improved renal fibrosis and inhibited inflammation in mice with lupus nephritis, which may play a role in inhibiting C3/MMPs and promoting prss-related protein expression.

**Supplementary Information:**

The online version contains supplementary material available at 10.1007/s00210-023-02883-x.

## Introduction

Systemic lupus erythematosus (SLE) is an autoimmune disease caused by environmental, genetic, endocrine, and other factors that cause chronic damage to various tissues and organs in the body (Zucchi et al. 2022). Lupus nephritis (LN) refers to SLE with different pathological types of immune damage in both kidneys accompanied by obvious clinical manifestations of renal damage (Anders et al. 2020). Animal models of SLE can be divided into two categories: spontaneous and inductive. MRL/LPR mice are representative animal models for studying the pathogenesis of SLE, with symptoms similar to those of human lupus in the natural state (Chen et al. 2021).

Human epididymis protein 4 (HE4), also known as WAP 4-disulfide core domain protein 2 (WFDC2), was first discovered in the distal epididymis and mature sperm, and then reported in the respiratory tract, genital tract, and kidney, successively, and is expressed at low levels in normal human cells (Shen Hongwei 2020; Ros et al. 2015; Karlsen et al. 2014). HE4 is believed to be a new tumor marker, especially in ovarian and lung cancers (Sieh et al. 2013; Dong et al. 2017). Studies have shown that elevated serum HE4 levels are observed in patients with diseases such as chronic kidney disease, renal fibrosis, renal failure, and heart failure (Nagy et al. 2012; Yuan and Li 2017; Chen et al. 2017; Boer et al. 2013), whereas HE4 is highly expressed and can be used as a biomarker to distinguish LN from SLE (Bedkowska et al. 2019; Yang et al. 2016). However, the mechanism by which HE4 regulates MMPs and proteins in lupus nephritis has not yet been reported. Therefore, in the present study, we used MRL/LPR transgenic mice at 10 weeks of age to explore the expression of MMPs/prss-related proteins in kidney tissues by injecting HE4 adenovirus into the renal pelvis. The present study analyzed the application value of HE4/MMPs/prss in the diagnosis of LN to provide a fundamental basis for the clinical diagnosis and treatment of LN.

## Materials and methods

### The construction and packaging of the shRNA adenovirus vector

The corresponding vectors were selected to design the PCR primers for the target fragment. Appropriate restriction enzymes were selected to digest the vector, and the purified linearized vector was recovered using agarose gel electrophoresis. The PCR reaction on the target fragment was conducted using the designed primers, and the correct size of the target fragment was recovered by agarose gel electrophoresis. The linearized vector and target fragment were ligated by homologous recombination or T4 ligation. After transformation into competent DH5a or stbl3 cells, the plates were coated with bacterial solution and cultured for 12–16 h. Monoclonal colonies were selected for verification via sequencing. The cloned samples with correct sequencing were used for plasmid extraction and the construction of the recombinant adenovirus vector.

### The packaging and quality analysis of adenovirus vector

The Adeasy adenovirus system was used for the virus packaging process. After high-purity endotoxin-free extraction of the plasmid vector, the plasmid was transfected into 293A cells using LipofiterTM transfection reagent. After virus production, a high-titer adenovirus preservation solution was obtained by virus amplification or purification using ultracentrivolution. Finally, the adenovirus indices were determined according to strict quality standards.

### The establishment of animal model and grouping

After adaptive feeding for 12 weeks, the model was established spontaneously and then randomly divided into two groups. The control and model groups were fed normally without any treatment. In the M+NC group, the empty shRNA vector (shRNA-NC) was injected into the renal pelvis on day 0 after modeling. In the M+ShRNA group, HE4 shRNA adenovirus was injected into the renal pelvis on day 0 after modeling. Urine was collected on days 0 and 5 after modeling for urine protein detection, and renal tissues were collected on day 5 at the end of the experiment for subsequent detection.

### HE staining

After collecting renal tissues from each animal, the tissues were rinsed with water for 2 h. After dehydration using different concentrations of ethanol solution, the tissues were dehydrated with xylene until transparent, embedded for 1 h, and sliced. Subsequently, the slides were roasted, dewaxed, hydrated, immersed in distilled water, and stained in hematoxylin aqueous solution for 3 min, followed by differentiation with hydrochloric acid ethanol differentiation solution for 15 s. After being slightly washed with water and blue-returning solution for 15 s, the slides were rinsed with water and stained with eosin (G1100, Solarbio, China) for 3 min. Images were obtained using an inverted microscope (CKX53, OLYMPUS, Japan).

### Immunofluorescence assay

Slides were rinsed in PBS for 1 h, followed by incubation with 10% goat serum overnight. The slides were then incubated with the primary antibody against HE4 (1:200, bs-4626R, Bioss, USA) or p-NF-κB p65 (1:100, AF2006, Affinity, USA) at 4 °C for 24 h, followed by washing with PBS. The secondary antibody (1:100, AS007, ABclonal, USA) was added and incubated at 4 °C for 24 h, followed by rinsing and staining with DAB dye. Finally, images were taken using a fluorescence microscope (BX53, OLYMPUS, Japan).

### Masson staining assay

Sections were immersed in Masson’s A solution at room temperature overnight, followed by staining with methyl green solution for 2–3 min. Slides were then immersed in Masson A solution and incubated in an oven at 65 °C for 30 min, followed by washing with tap water for 30 s until the yellow color of the tissues faded. Simultaneously, Masson D and Masson F solutions were preheated in a 65 °C oven. Masson B solution and Masson C solution were mixed in equal volumes, and the sections were immersed in the mixture for 1 min and washed slightly with running water, followed by differentiation with 1% hydrochloric alcohol for approximately 1 min until the nuclei were gray-black and the background was almost colorless or light gray. The sections were then slightly washed with tap water, and the excess water on the sections was slightly drained, followed by staining with Masson D solution for 6 min. Subsequently, the sections were slightly drained and soaked in Masson’s E solution for approximately 1 min. After the sections were slightly drained in Masson’s E solution, they were directly transferred into Masson’s F solution and stained for 2–30 s. The sections were rinsed with 1% glacial acetic acid in three consecutive cylinders and differentiated for approximately 8 s in each cylinder, followed by successive dehydration by three consecutive cylinders of absolute ethanol for approximately 5 s, 10 s, and 30 s, respectively, and finally made transparent by two cylinders of xylene for 5 min each time. The slices were sealed with neutral gum, washed, dried, and examined under an inverted microscope (CKX53, OLYMPUS, Japan).

### Immunohistochemical assay

Levels of renal injury marker proteins were detected using the immunohistochemical assay. Renal tissue sections were deparaffinized and hydrated, and then antigen retrieval was performed using citrate buffer. After blocking with 5% BSA, sections were incubated with rabbit anti-β2-MG antibody (1:200, 13511-1-AP, Proteintech, USA), rabbit anti-NGAL antibody (1:200, PB9609, BOSTER, USA), and rabbit anti-Kim-1 antibody (1:200, bs-21420R, Bioss, USA) overnight at 4°C. The sections were then incubated with the goat anti-rabbit secondary antibody (1:100, ab150077, Abcam, USA) for 30 min at room temperature. After DAB staining, hematoxylin counterstaining, dehydration, and transparency, the sections were sealed and observed under a microscope (BX43, Olympus).

### The detection of the total urine protein and renal function

The detection was conducted according to the manufacturer’s instructions for the urine total protein detection kit (colorimetric method), urea detection kit (urease-glutamate dehydrogenase method), uric acid detection kit (TBHBA method), and creatinine detection kit (enzymatic method).

### Western blotting assay

The BCA kit (Cwbio, Jiangsu, China) was used to quantify the protein isolated from renal tissues, followed by separation using 12% SDS-PAGE. The separated proteins were transferred from the gel to a PVDF membrane, which was further incubated with 5% skim milk. The membranes were then incubated with primary antibodies against C3 (1:500, DF13224, Affinity, USA), HE4 (1:500, DF8160, Affinity, USA), MMP9 (1:500, AF5228, Affinity, USA), Prss35 (1:500, Ab151970, Abcam, UK), Prss23 (1:500, NBP2-93072, Novus, USA), MMP2 (1:500, 10373-2-AP, Proteintech, USA), P-P65 (1:500, AF2006, Affinity, USA), and β-actin (1:2000, HC201, TransGen Biotech, China). The secondary antibody (1:2000, GB23301; Servicebio, China) was subsequently added and incubated for 90 min. Finally, ECL reagent was added to expose the bands, which were further quantified using the ImageJ software.

### Statistical analysis

Mean±SD was utilized to present data, which was analyzed using the one-way ANOVA method with the software of GraphPad Prism 8.0.1 software. *P*<0.05 was considered a statistically significant difference.

## Results

### Expression level of HE4 in renal tissues of different groups

**Fig. 1:**
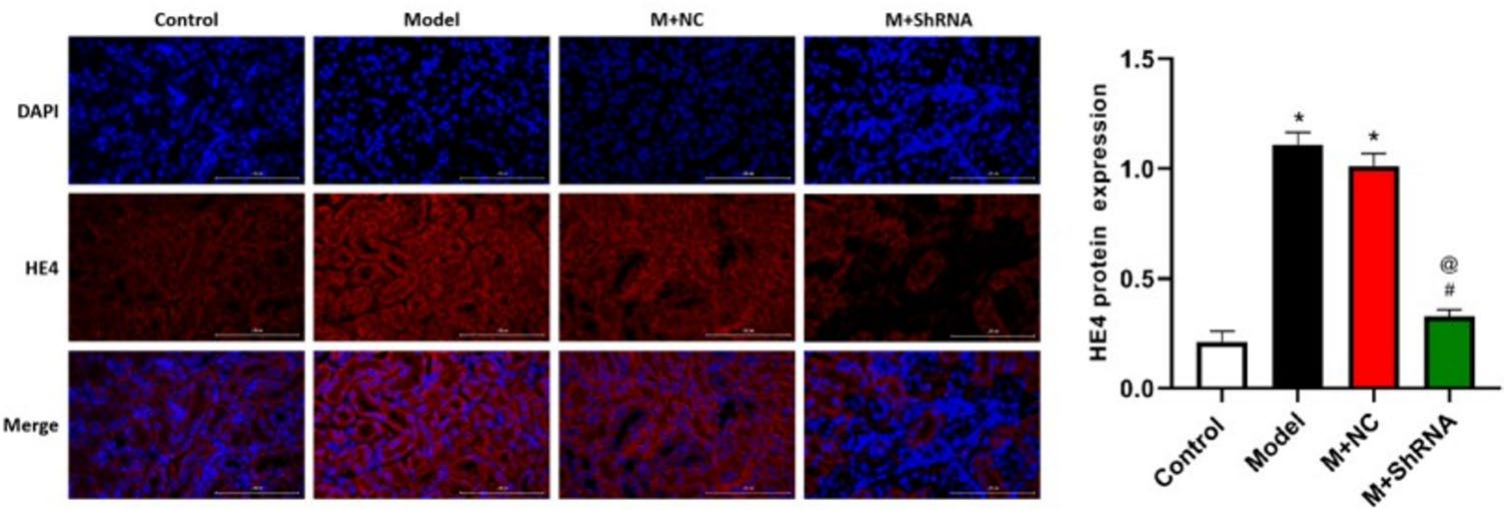
MRL/LPR mice were injected with the adenovirus containing the shRNA-NC or HE4 shRNA. The expression level of HE4 in renal tissues was detected by the immunofluorescence assay (*n*=3, ×400, *p<0.05 vs. control, #*p*<0.05 vs. model, @*p*<0.05 vs. M+ NC)

The expression of HE4 protein expression in renal tissues was detected by immunofluorescence assay after injection of HE4 shRNA adenovirus into the renal pelvis. DAPI-stained nuclei were blue under ultraviolet excitation, and the positive expression of HE4 corresponded to fluorescein-labeled red light. As shown in Fig. 1, after tail vein injection of shRNA, HE4 was significantly downregulated, suggesting successful knockdown of HE4 in MRL/LPR mice.

### The impact of HE4 on the location and expression of p-NF-κB p65 in the renal tissues of MRL/LPR mice

**Fig. 2:**
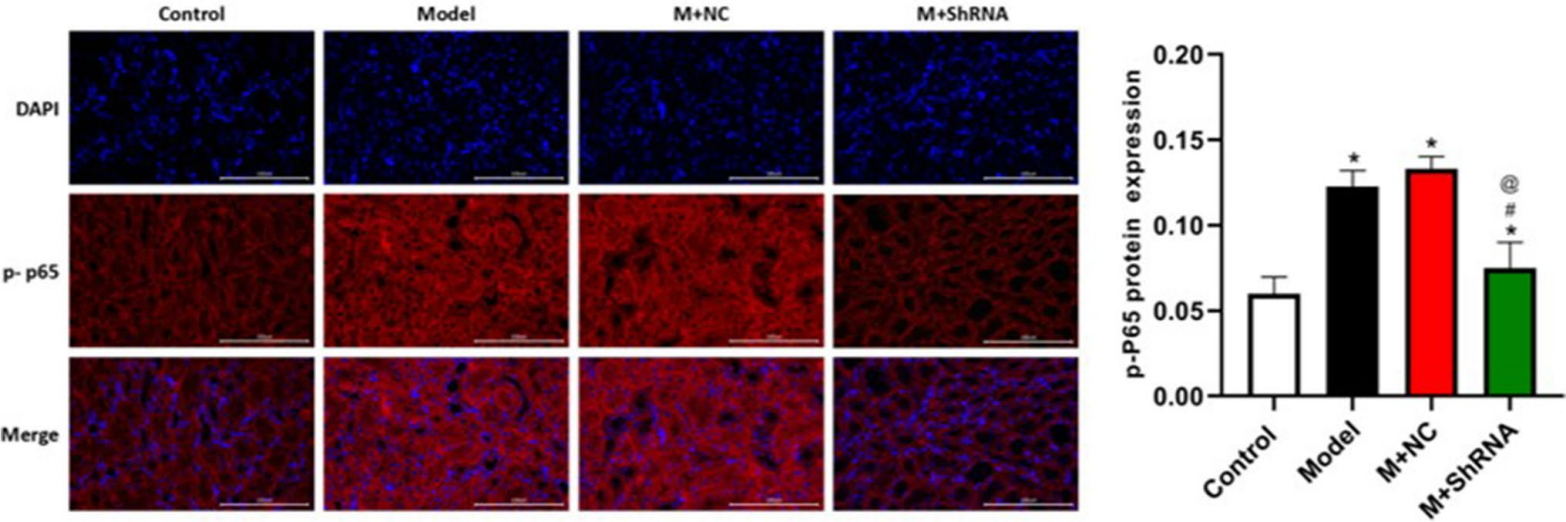
MRL/LPR mice were injected with the adenovirus containing the shRNA-NC or HE4 shRNA. The location and expression of p-NF-κB p65 were determined by the immunofluorescence assay (*n*=3, ×400, *p<0.05 vs. control, # *p*<0.05 vs. model, @*p*<0.05 vs. M+ NC)

The expression and localization of p-NF-κB p65 protein in renal tissue samples were detected by immunofluorescence. As shown in Fig. 2, the p-NF-κB p65 protein was located in the cytoplasm, and compared to control, the expression of p-NF-κB p65 in the model group was significantly increased, which was markedly decreased by the knockdown of HE4.

### The impact of HE4 on pathological changes and fibrosis state in renal tissues of MRL/LPR mice

**Fig. 3.**
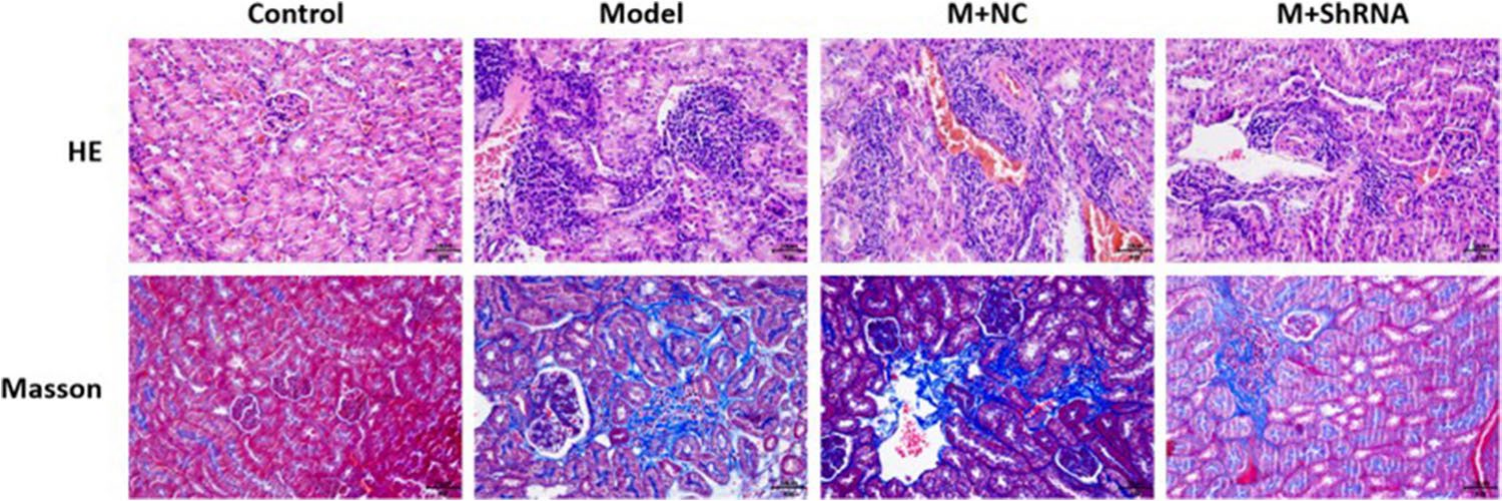
MRL/LPR mice were injected with the adenovirus containing the shRNA-NC or HE4 shRNA. The pathological changes and fibrosis state in renal tissues were evaluated by HE staining and Masson staining assay, respectively (*n*=3, ×400)

As shown in Fig. 3, in the control group, no obvious lymphocytic invasion and apparent volume increase or necrosis were observed in the glomerulus, with well-defined capillary loops of the glomerulus and lumen of thin normal endothelial cells and mesangial cells. In the model group, a marked increase in the number of glomeruli, hyperplasic basement membrane, severe infiltration of inflammatory cells in the renal tubules and glomeruli, obvious necrosis in the glomeruli, and severe bleeding were observed. After administration of the HE4 shRNA adenovirus vector, the repaired structure of renal tubules and glomeruli and improved infiltration of inflammatory cells were observed. Masson staining was used to evaluate the fibrotic state in renal tissues, with renal muscle fibers stained red and collagen fibers stained blue. In the model group, severe diffuse proliferation of the glomerular basement membrane and mesangium, stenosis and occlusion of the capillary lumen, and increased expression of collagen fibers were observed. After administration of the HE4 shRNA adenovirus vector, fibrosis progression was repressed, and renal injury was repaired.

### The impact of HE4 on the expression of marker proteins of renal injury in renal tissues of MRL/LPR mice

**Fig. 4.**
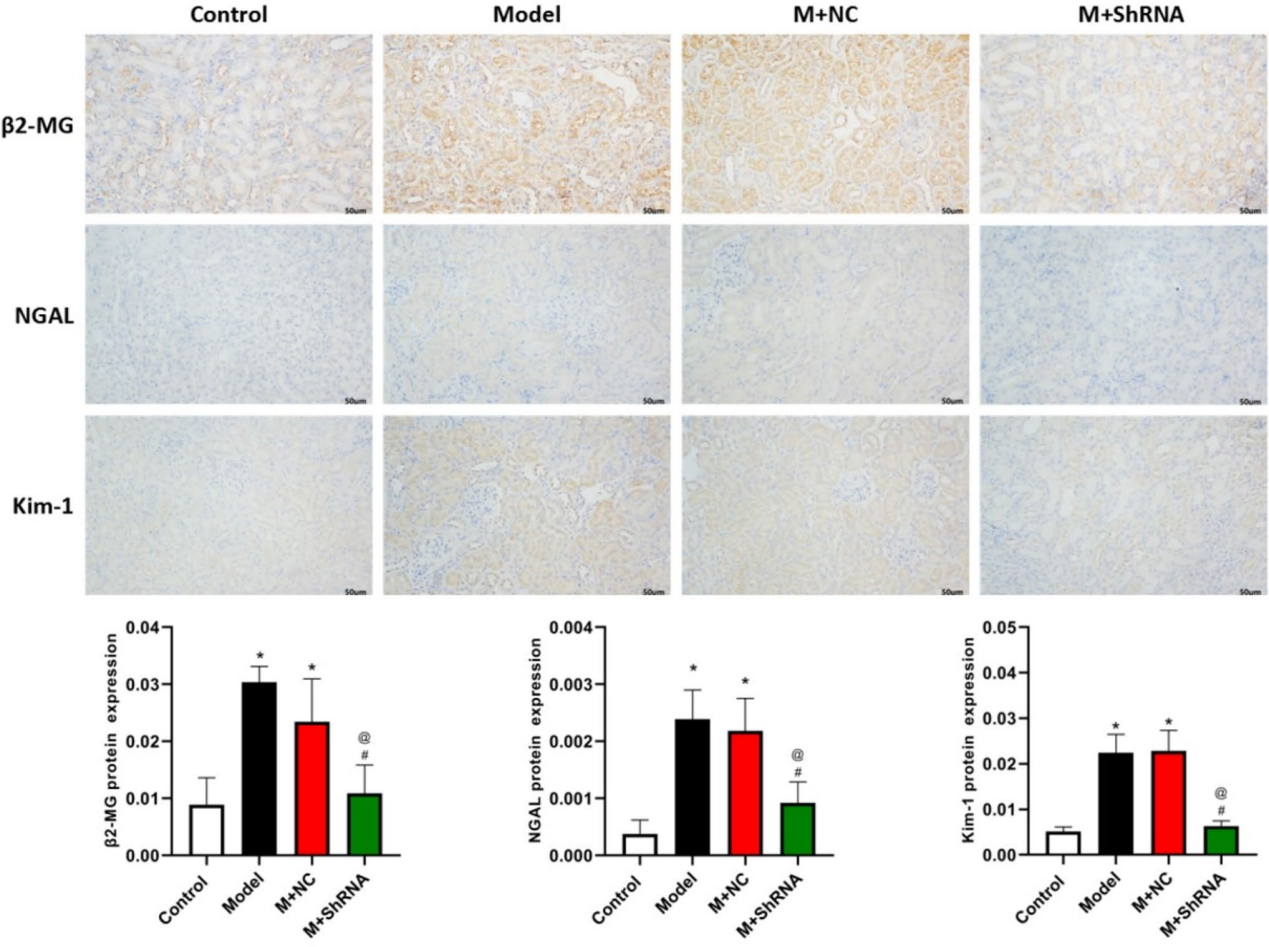
MRL/LPR mice were injected with the adenovirus containing the shRNA-NC or HE4 shRNA. The expression of β2-MG, NGAL, and Kim-1 in renal tissues was detected using the immunohistochemical assay (*n*=3, ×400, *p<0.05 vs. control, # *p*<0.05 vs. model, @*p*<0.05 vs. M+ NC)

To further verify the repair effect of silencing HE4 on lupus nephritis-induced renal injury, the immunohistochemical method was utilized to detect the expression of marker proteins β-2-microglobulin, NGAL, and Kim-1 in kidney tissue. As shown in Fig. 4, compared to control, levels of β-2-MG, NGAL, and Kim-1 in the model group were significantly increased in renal tissues, which were sharply reduced, indicating that renal injury had been repaired by silencing HE4.

### The impact of HE4 on the urine protein level in MRL/LPR mice

**Fig. 5.**
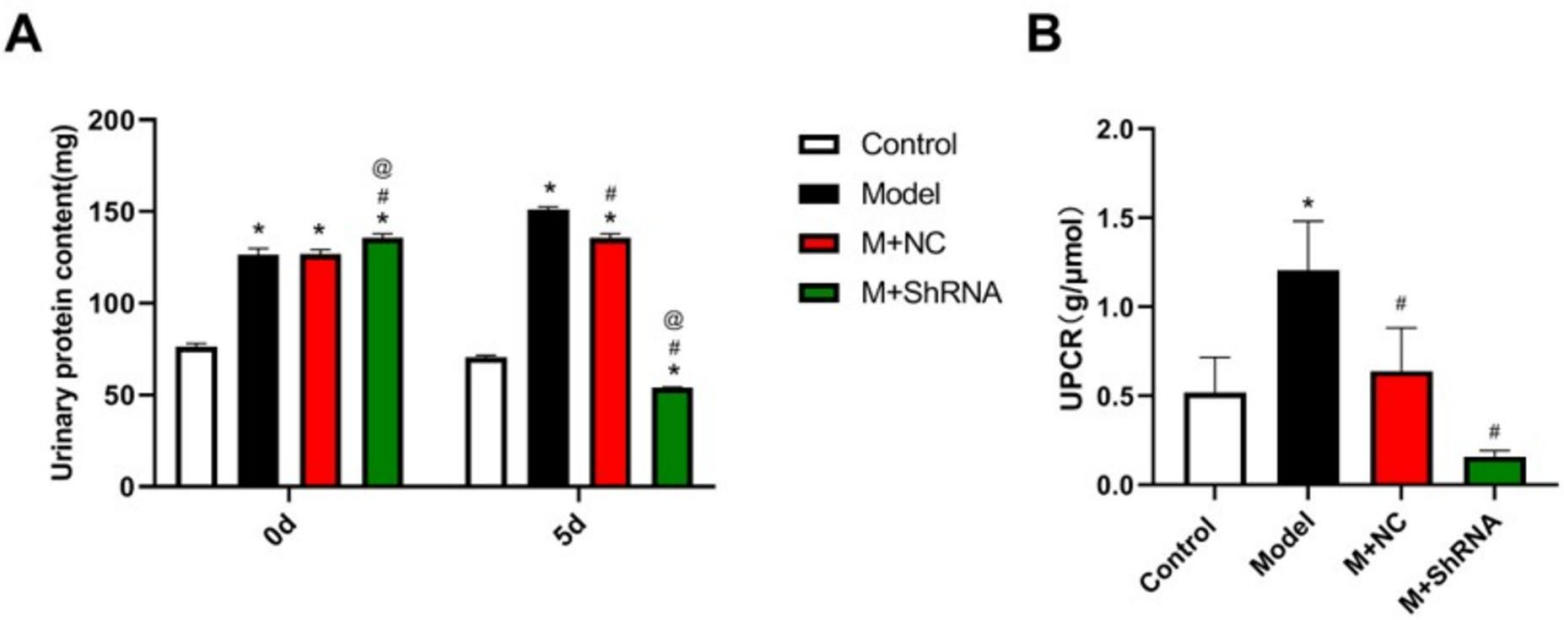
MRL/LPR mice were injected with the adenovirus containing the shRNA-NC or HE4 shRNA. **A** The urine protein level on days 0 and 5 was detected using the colorimetric method. **B** The UPCR value in renal tissues was determined (*n*=3, *p<0.05 vs. control, # *p*<0.05 vs. model, @*p*<0.05 vs. M+ NC)

Urinary protein level is the most important marker for the evaluation of LN, and 24-h urinary protein quantification is the gold standard for the diagnosis of rational urinary protein by domestic and foreign scholars. The difference in the level of urine protein in MRL/LPR mice before and after administration of the HE4 shRNA adenovirus vector was detected in the present study. As shown in Fig. 5A, on day 0, a dramatically increased level of urine protein was observed in the model, M+NC, and M+ shRNA groups, with significant differences, indicating that the lupus nephritis model was spontaneously established in 12-week-old MRL/LPR transgenic mice. On day 5, compared to the control group, markedly elevated urine protein levels were observed in the model and M+NC groups. Compared to the model and M+NC groups, the urine protein level in the M+ shRNA group was dramatically reduced.

In addition, the differences in the urine protein level to creatinine ratio (UPCR) were compared. As shown in Fig. 5B, UPCR in the model group was significantly higher than that in the control group, which was greatly repressed in the M+ shRNA group, suggesting that the progression of LN disease was improved by the knockdown of HE4.

### The impact of HE4 on the expression level of C3, HE4, MMP2, MMP9, p-P65, prss23, and prss35 in renal tissues in MRL/LPR mice

**Fig. 6.**
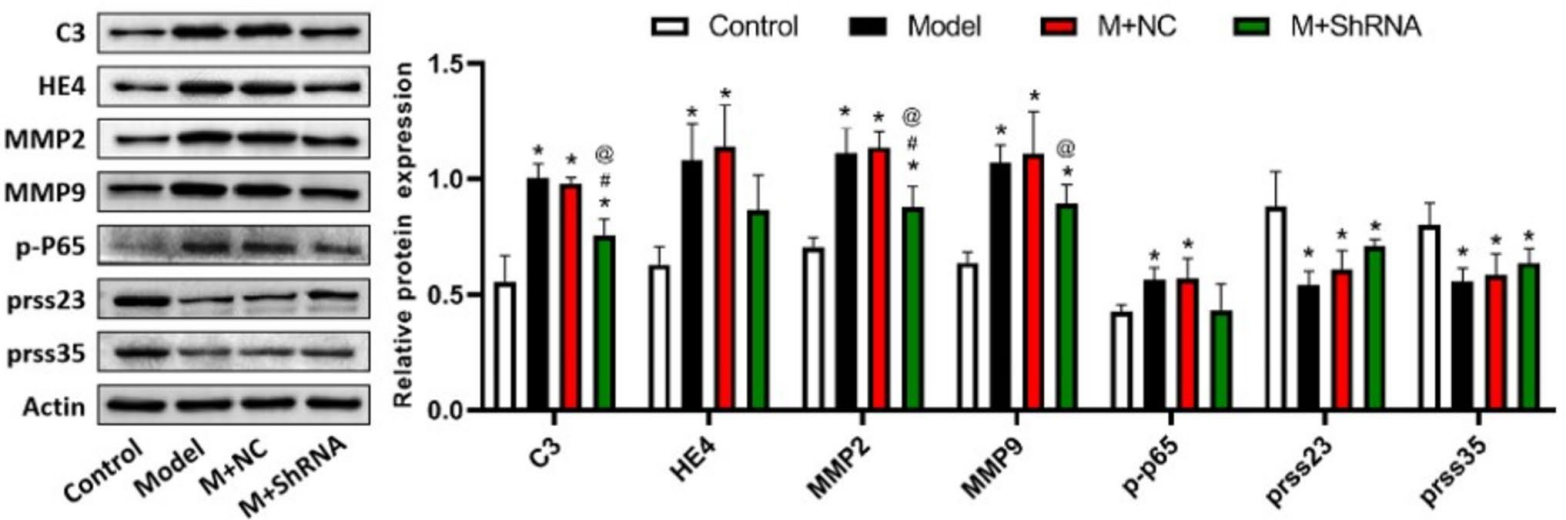
MRL/LPR mice were injected with the adenovirus containing the shRNA-NC or HE4 shRNA. The protein level of C3, HE4, MMP2, MMP9, p-P65, prss23, and prss35 in renal tissues was determined by the Western blotting assay (*n*=3, *p<0.05 vs. control, #*p*<0.05 vs. model, @*p*<0.05 vs. M+ NC)

Lastly, related protein levels in renal tissues were detected by western blotting. As shown in Fig. 6, compared to the control, C3, HE4, MMP2, MMP9, and p-P65 were dramatically upregulated, while prss23 and prss35 were greatly downregulated in the model group. Compared to the M+ NC group, no significant changes in the protein level of p-P65 were observed in the M+ shRNA group. Compared to the model and M+NC groups, C3, MMP2, and MMP9 were markedly downregulated, whereas prss23 and prss35 were slightly upregulated in the M+ ShRNA group.

## Discussion

Herein, HE4 was found markedly upregulated in renal tissues of the SLE mouse model, implying that HE4 might be a potential pathogenic protein for renal fibrosis in SLE. NF-κB is an important pro-inflammatory signaling involved in multiple diseases (Wang and Shen 2022). After the internal stimulation, the activated state of NF-κB (p65) will be phosphorylated and transferred into the nucleus to activate the transcription of multiple inflammatory factors (Barnabei et al. 2021). Herein, in the renal tissues of SLE mouse model, pathological changes observed in renal tissues were accompanied by markedly upregulated p-NF-κB p65, indicating an activation of the NF-κB signaling in injured renal tissues of SLE mice. Furthermore, the pathological changes were alleviated by silencing HE4, accompanied by the largely reduced p-NF-κB p65 level, implying that silencing HE4 might protect renal injury by inactivating NF-κB.

NAG, as one of the lysosomal enzymes, is present in various tissues and organs, especially with high expression in the proximal tubule. Due to its high molecular weight, it is difficult for NAG to penetrate the glomerulus and thus cannot be increased through non-renal sources (Vibulcharoenkitja et al. 2021). NAG can be cleared from the urine through exocytosis of renal tubular epithelial cells, resulting in only trace amounts of NAG in the urine. However, after kidney tubular damage, NAG exhibits high activity in the urine (Safaeian et al. 2021). β2-MG is a small molecular weight protein with a molecular weight of 11.8 kDa that can be filtered by the glomerulus and almost completely reabsorbed and decomposed by the proximal tubule. Under pathological conditions, the proximal tubule is damaged, leading to dysfunctional reabsorption and increased urinary β2-MG content, making it a commonly used clinical evaluation index for renal tubular function (Loureiro and Faisca 2020). NGAL is a secretory protein, and NGAL in plasma can be filtered by the glomerulus and reabsorbed by the proximal tubule, resulting in very low concentrations in urine that are difficult to detect (Romejko et al. 2023). Under pathological conditions, NGAL synthesis in the proximal tubule increases and its reabsorption decreases, ultimately leading to elevated urinary NGAL expression under dual effects (Brewin et al. 2022). Herein, β2-MG, NGAL, and NAG levels were markedly increased in renal tissues of SLE mice, which were sharply decreased by silencing HE4, further confirming the protective effect of silencing HE4 against the renal injury in SLE mice.

Urine protein levels are essential for LN diagnosis, disease activity monitoring, and prognosis evaluation in patients with SLE. Another clinical method to measure urinary protein is to calculate the value of UPCR because of the similar dilution extent of urine protein and creatinine in the urine, which can be corrected for changes in urine concentration due to dehydration. UPCR is more reliable for reflecting results of 24-h urinary protein quantification, which is also recommended by the American College of Rheumatology (ACR) and the European League of Rheumatology (EULAR) (Chedid et al. 2020; Huang et al. 2020). The results of a meta-analysis (Medina-Rosas et al. 2016) showed that UPCR showed a good correlation with 24-h urinary protein, and it has been proven that UPRC in morning urine can better evaluate the urinary protein level of LN patients (Zhang Huayong et al. 2016). According to the results of the present study, in 12-week-old MRL/LPR mice, the urine protein and UPCR were dramatically higher than those in the wild-type mice, which were markedly reduced by silencing HE4, indicating the renal function of SLE mice was improved by silencing HE4.

Previous studies have shown that the pathogenesis of LN is closely related to complement factors (Gasparotto et al. 2020). C3 is the most abundant complement component in the serum, and the deposition of C3 is a characteristic of LN, which is an important part of the classical, alternative, and complement mannose pathways. Complement factor C3 is heavily deposited in the glomerulus and induces the proliferation of glomerular mesangial cells, exacerbating the process of renal fibrosis and eventually leading to nephropathy (Li et al. 2022; Shi et al. 2019). By stimulating the formation of extracellular matrix (ECM) through the TGF-β/Smads or Akt pathway, C3 induces renal tubular epithelial cells to transform into myofibroblasts and aggravates the renal interstitial fibrosis process (Wang et al. 2020). Furthermore, renal fibrosis was alleviated in C3-knockdown UUO mice, suggesting that C3 is involved in the pathogenesis of renal fibrosis (Cui Jiong et al. 2019). Recently, researchers found that complement factor inhibitors can effectively improve urine protein and renal function in MRL/LPR mice. For example, LNP023, an alternative complement factor B(CFB) pathway inhibitor, inhibits activation of the alternative complement pathway. Compared with the MRL/LPR group, the LNP023 group showed reduced lupus-like symptoms, improved renal function, and reduced C3 deposition in the serum, renal tissues, and liver tissues. Moreover, LNP023 alleviated pathological damage in the kidneys of MRL/LPR mice (Chen et al. 2022). In the present study, it was confirmed that C3 levels were significantly increased in mice with lupus nephritis, which was dramatically repressed by the knockdown of HE4, indicating that a regulatory relationship might exist between HE4 and C3.

Matrix metalloproteinases (MMPs) are a family of endopeptidases that play specific roles in ECM degradation and turnover (Almeida et al. 2022). For example, in patients with endometriosis, the expression of MMP-2 and MMP-9 in the endometrium is increased, which in turn promotes the rupture of peritoneal ECM and the establishment of peritoneal endometriosis foci through the increase in MMP activity in the endometrium (Barbe et al. 2020). Similarly, Wang et al. (Wang et al. 2019) detected high levels of HE4 in the serum of patients with pulmonary fibrosis (CF). The expression of NF-κB p-P65, MMP2, and MMP9 was elevated by the overexpression of HE4, which was repressed by the knockdown of HE4, accompanied by the alleviation of fibrosis, suggesting that the normal EMC was disrupted by the HE4/MAPK/MMPs signaling cascade to aggravate the progression of CF. In our study, it was found that the downregulation of MMP2, MMP-9, and p-P65 in renal tissues of LN mice was induced by the knockdown of HE4, which was consistent with previous results.

Prss23 and prss35 belong to the tryptic class of serine proteases and were originally identified as homologous proteases in mouse ovaries (Xiong et al. 2023; Wang et al. 2023). Studies have shown that prss35 and prss23 function as HE4 targets in renal fibrosis. LeBleu et al. (LeBleu et al. 2013) found that HE4 was significantly upregulated in fibrotic renal tissues in three different mouse models of kidney disease and that the use of HE4 neutralizing antibodies accelerated the degradation of type I collagen and inhibited fibrosis. These results indicate that HE4 specifically inhibits prss35 and prss23 serine protease activities and their ability to degrade type I collagen, and acts as a potential pan-serine protease inhibitor. In our study, we found that the expression of prss35 and prss23 was elevated by HE4 knockdown, but the difference was not significant.

Collectively, HE4 was highly expressed in the mice with lupus nephritis. Downregulation of HE4 showed a promising effect in improving lupus nephritis and renal fibrosis in mice, which might be associated with the inhibition of C3/MMPs and promotion of the expression of prss-related proteins.

### Supplementary Information

Below is the link to the electronic supplementary material.

Supplementary file1 (ZIP 2112 KB)

## Data Availability

The datasets used and/or analyzed during the current study are available from the corresponding author on reasonable request.
