# Supplementary figures and images for: Silencing HE4 alleviates the renal fibrosis in lupus nephritis mice by regulating the C3/MMPs/prss axis
Source: Naunyn Schmiedebergs Arch Pharmacol. 2023 Dec 29;397(7):4823–31. doi: 10.1007/s00210-023-02883-x (PMC11166803; doi:10.1007/s00210-023-02883-x)

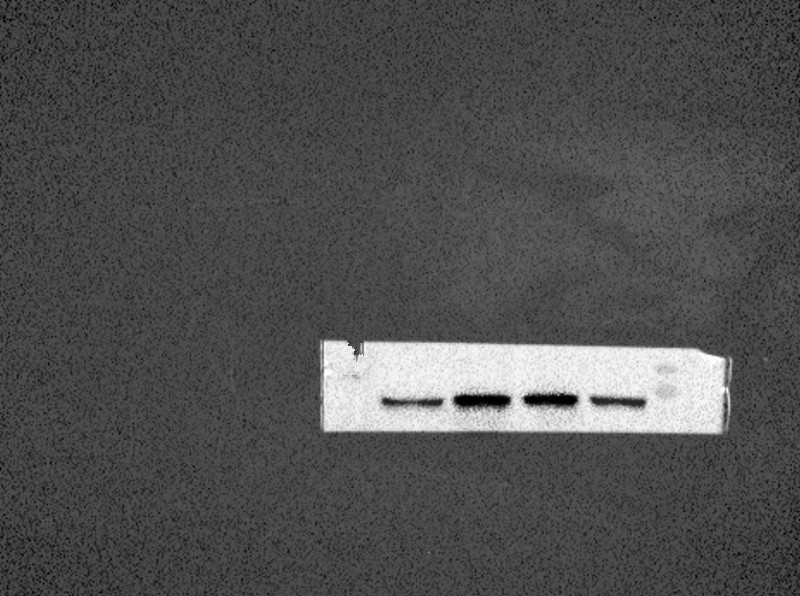

Supplement: Supplementary file 1 — Supplementary file1 (ZIP 2112 KB) [file 210_2023_2883_MOESM1_ESM.zip › full uncropped Gels and Blots image/C3/1.jpg]

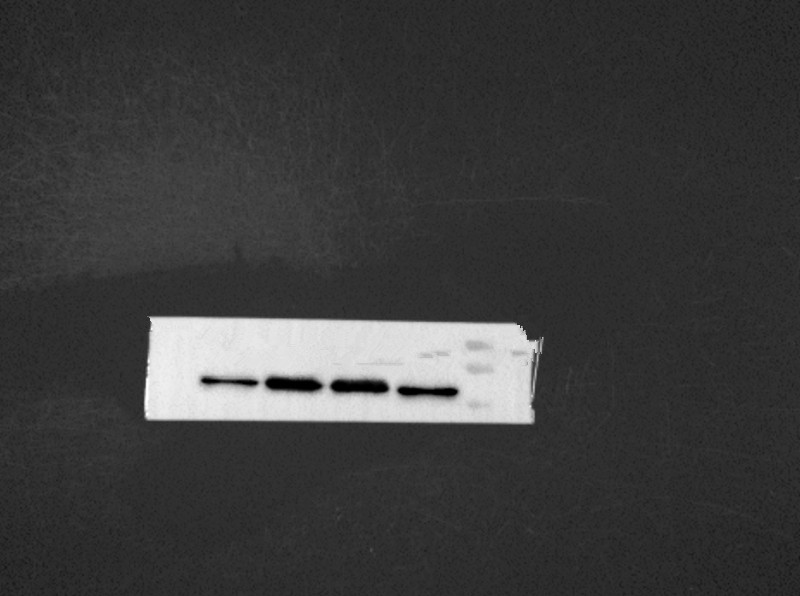

Supplement: Supplementary file 1 — Supplementary file1 (ZIP 2112 KB) [file 210_2023_2883_MOESM1_ESM.zip › full uncropped Gels and Blots image/C3/2.jpg]

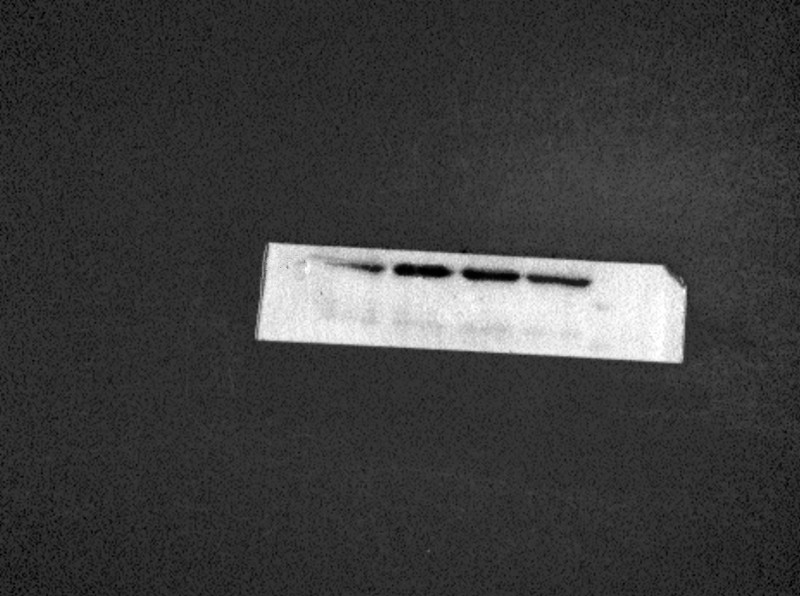

Supplement: Supplementary file 1 — Supplementary file1 (ZIP 2112 KB) [file 210_2023_2883_MOESM1_ESM.zip › full uncropped Gels and Blots image/C3/3.jpg]

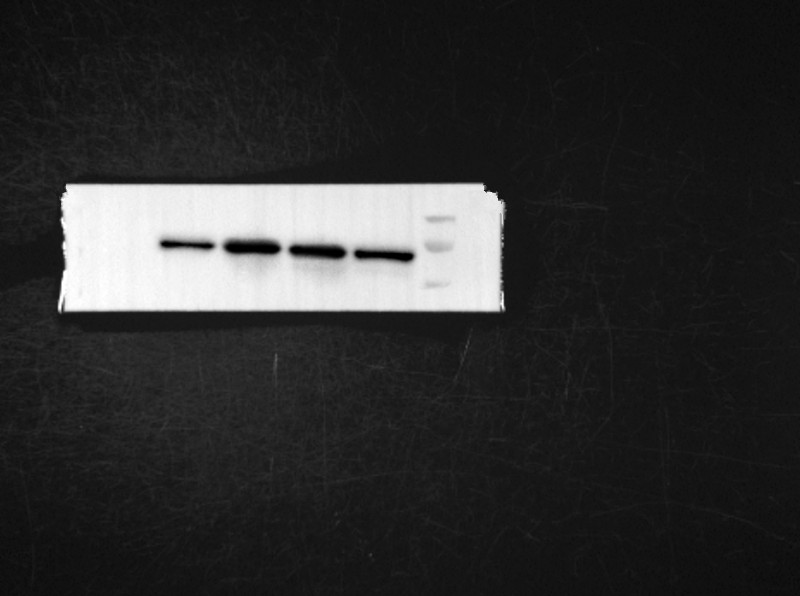

Supplement: Supplementary file 1 — Supplementary file1 (ZIP 2112 KB) [file 210_2023_2883_MOESM1_ESM.zip › full uncropped Gels and Blots image/HE4/1.jpg]

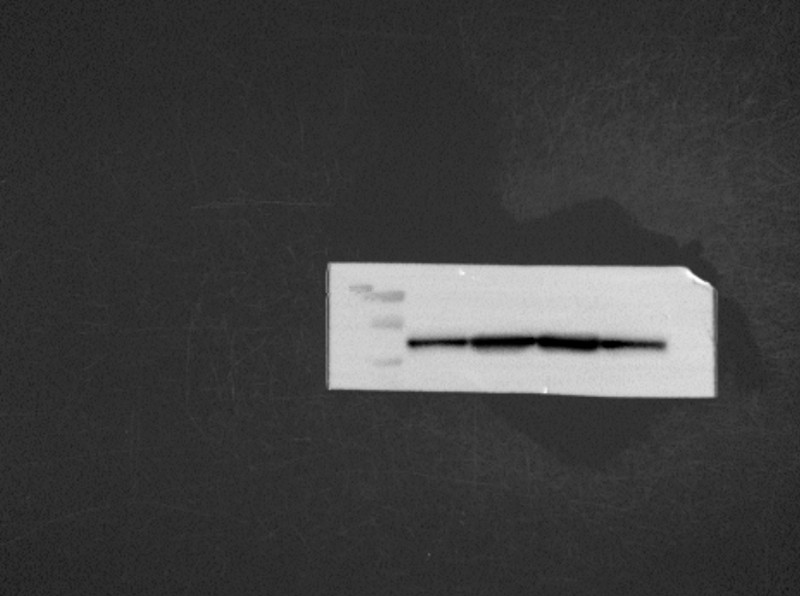

Supplement: Supplementary file 1 — Supplementary file1 (ZIP 2112 KB) [file 210_2023_2883_MOESM1_ESM.zip › full uncropped Gels and Blots image/HE4/2.jpg]

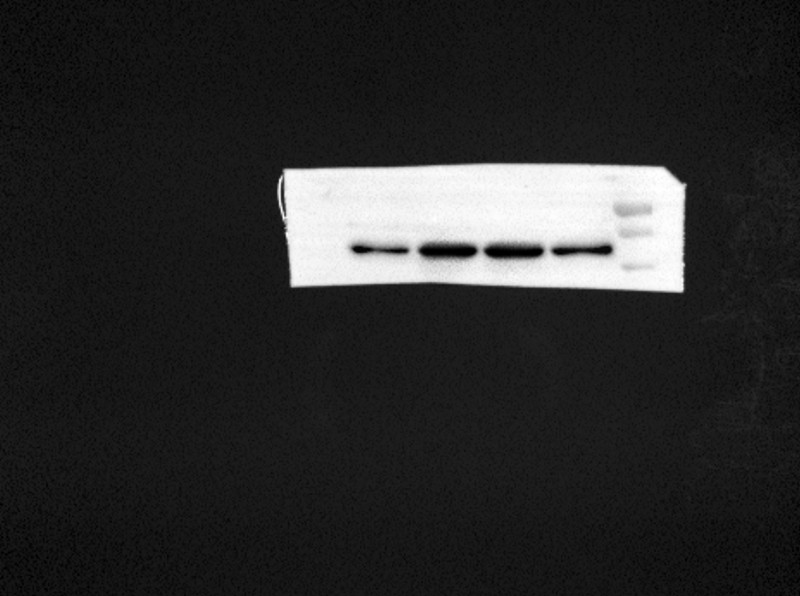

Supplement: Supplementary file 1 — Supplementary file1 (ZIP 2112 KB) [file 210_2023_2883_MOESM1_ESM.zip › full uncropped Gels and Blots image/HE4/3.jpg]

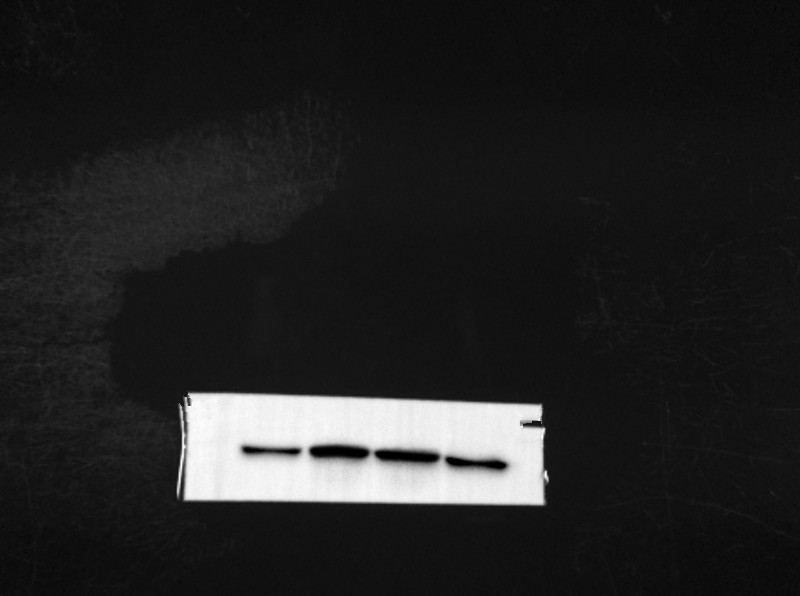

Supplement: Supplementary file 1 — Supplementary file1 (ZIP 2112 KB) [file 210_2023_2883_MOESM1_ESM.zip › full uncropped Gels and Blots image/MMP2/1.jpg]

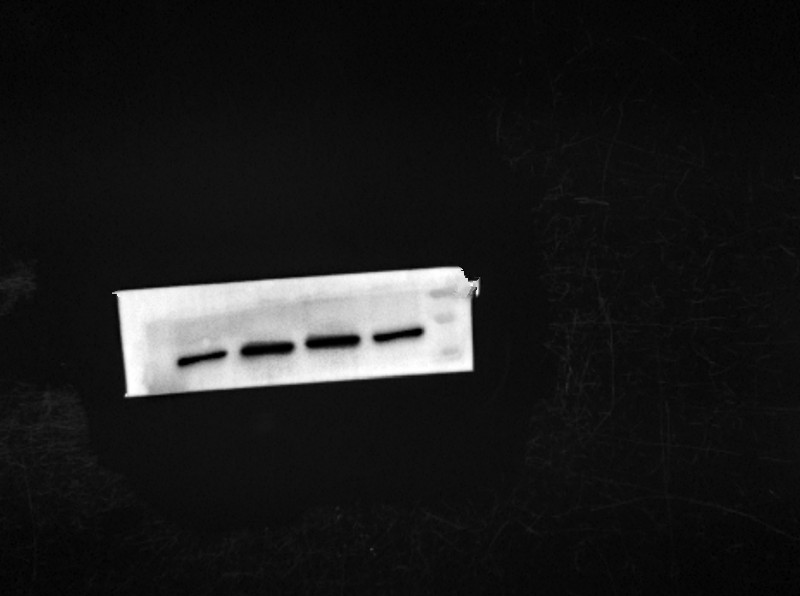

Supplement: Supplementary file 1 — Supplementary file1 (ZIP 2112 KB) [file 210_2023_2883_MOESM1_ESM.zip › full uncropped Gels and Blots image/MMP2/2.jpg]

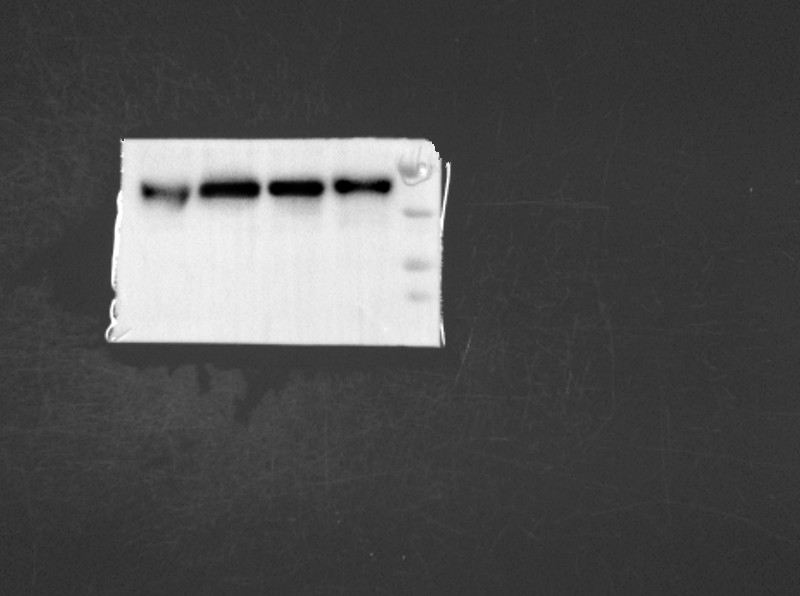

Supplement: Supplementary file 1 — Supplementary file1 (ZIP 2112 KB) [file 210_2023_2883_MOESM1_ESM.zip › full uncropped Gels and Blots image/MMP2/3.jpg]

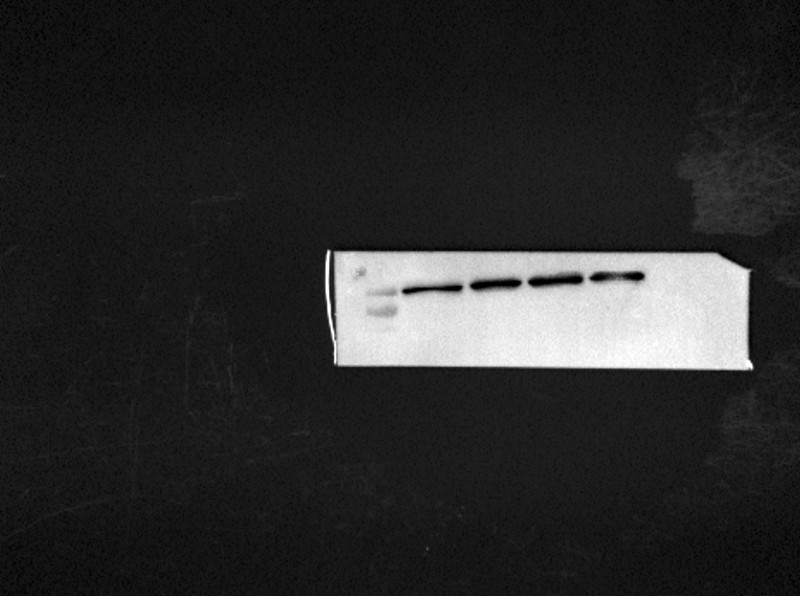

Supplement: Supplementary file 1 — Supplementary file1 (ZIP 2112 KB) [file 210_2023_2883_MOESM1_ESM.zip › full uncropped Gels and Blots image/MMP9/1.jpg]

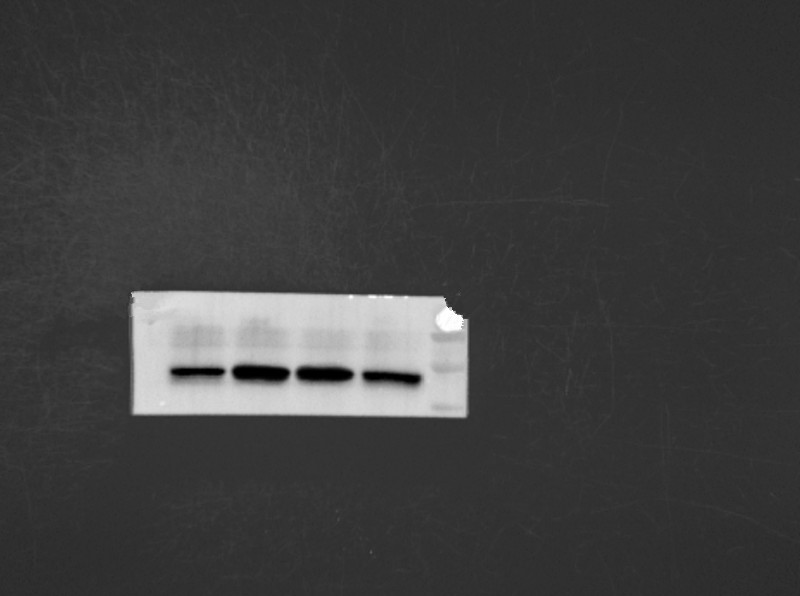

Supplement: Supplementary file 1 — Supplementary file1 (ZIP 2112 KB) [file 210_2023_2883_MOESM1_ESM.zip › full uncropped Gels and Blots image/MMP9/2.jpg]

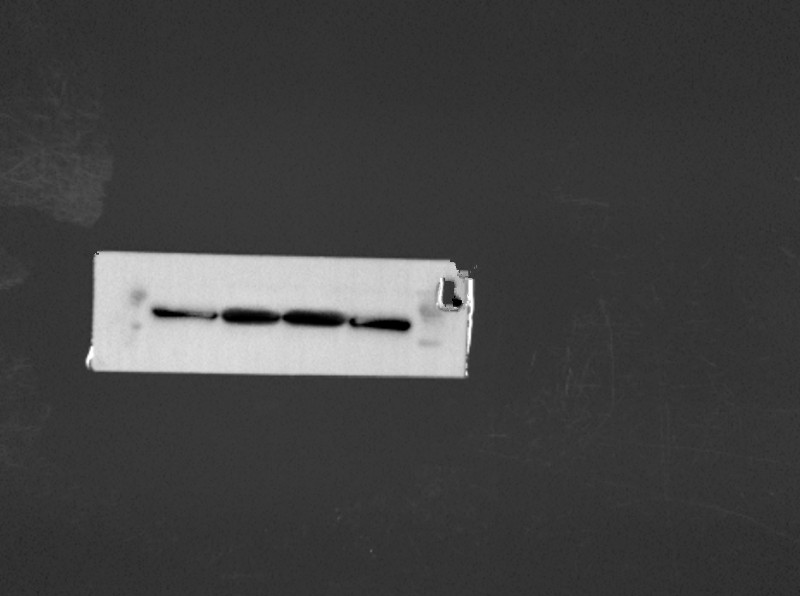

Supplement: Supplementary file 1 — Supplementary file1 (ZIP 2112 KB) [file 210_2023_2883_MOESM1_ESM.zip › full uncropped Gels and Blots image/MMP9/3.jpg]

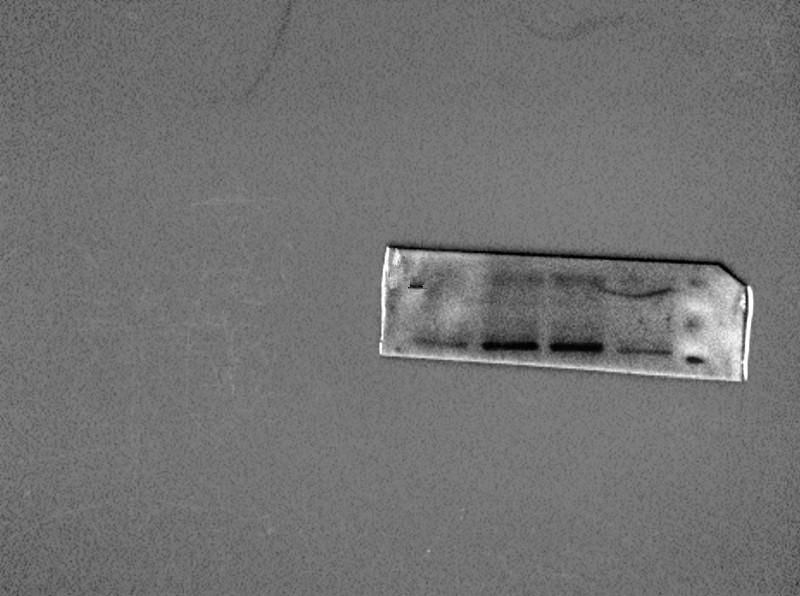

Supplement: Supplementary file 1 — Supplementary file1 (ZIP 2112 KB) [file 210_2023_2883_MOESM1_ESM.zip › full uncropped Gels and Blots image/p-p65/1.jpg]

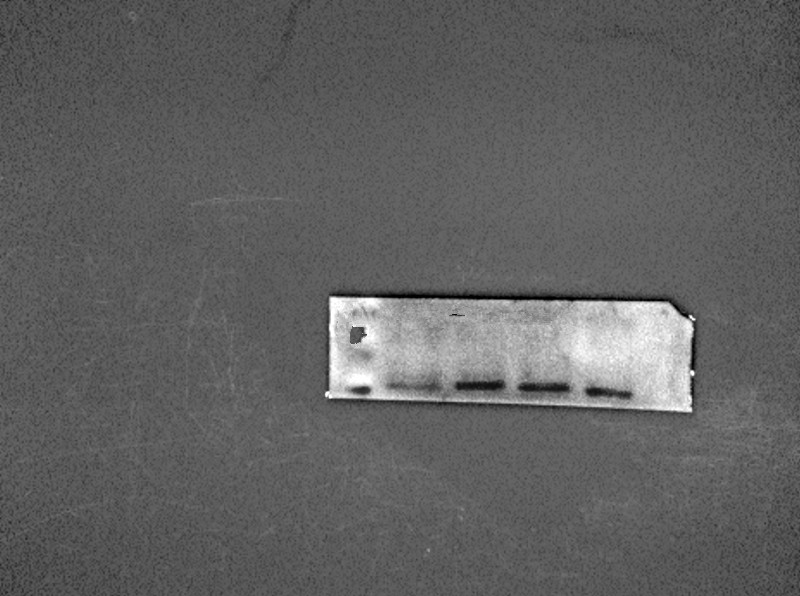

Supplement: Supplementary file 1 — Supplementary file1 (ZIP 2112 KB) [file 210_2023_2883_MOESM1_ESM.zip › full uncropped Gels and Blots image/p-p65/2.jpg]

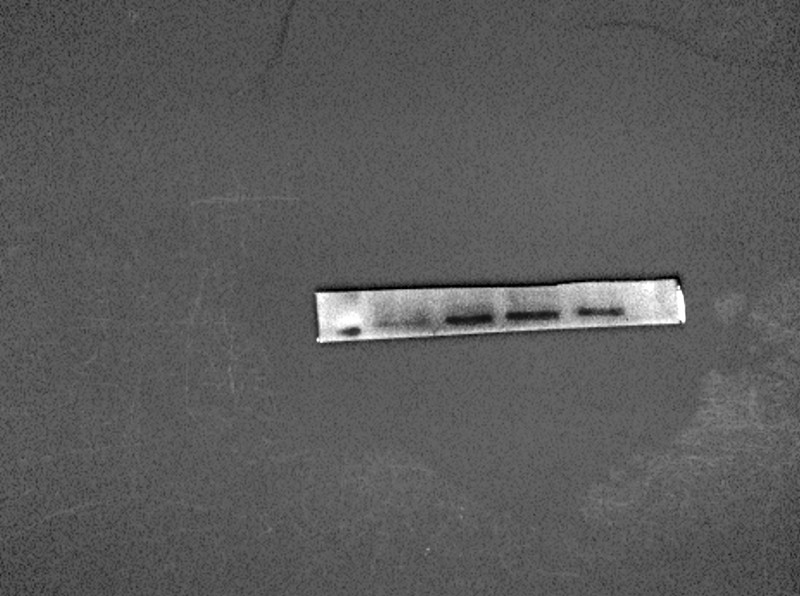

Supplement: Supplementary file 1 — Supplementary file1 (ZIP 2112 KB) [file 210_2023_2883_MOESM1_ESM.zip › full uncropped Gels and Blots image/p-p65/3.jpg]

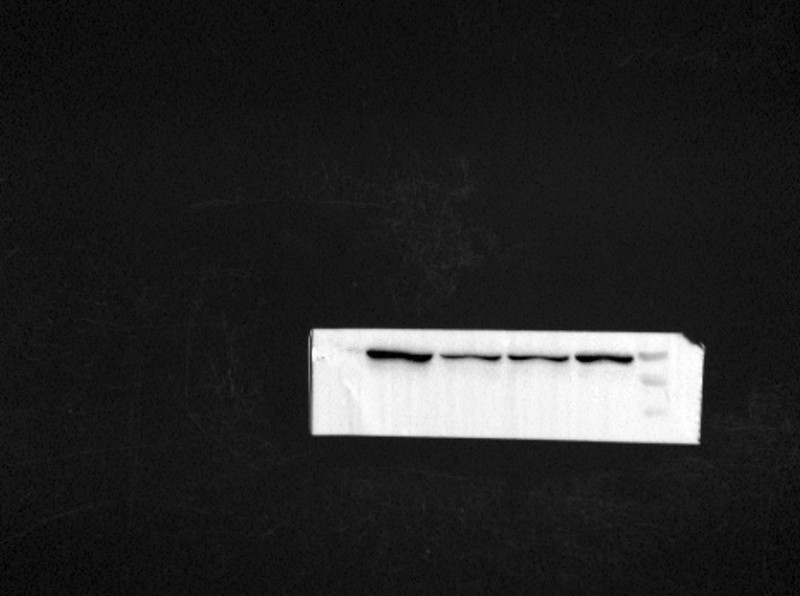

Supplement: Supplementary file 1 — Supplementary file1 (ZIP 2112 KB) [file 210_2023_2883_MOESM1_ESM.zip › full uncropped Gels and Blots image/prss23/1.jpg]

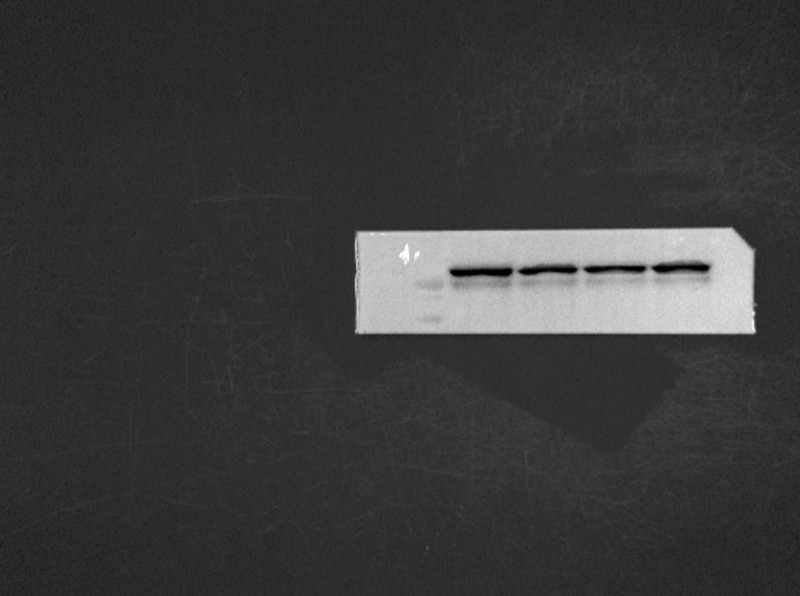

Supplement: Supplementary file 1 — Supplementary file1 (ZIP 2112 KB) [file 210_2023_2883_MOESM1_ESM.zip › full uncropped Gels and Blots image/prss23/2.jpg]

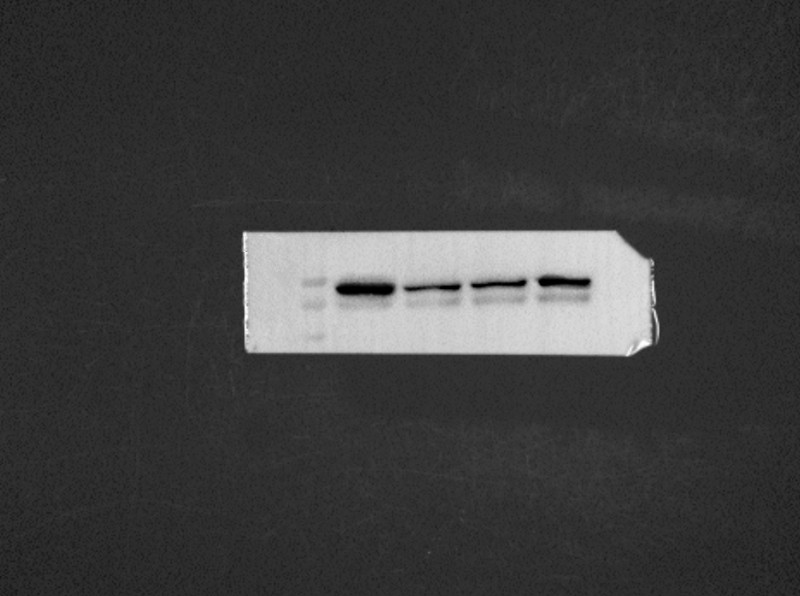

Supplement: Supplementary file 1 — Supplementary file1 (ZIP 2112 KB) [file 210_2023_2883_MOESM1_ESM.zip › full uncropped Gels and Blots image/prss23/3.jpg]

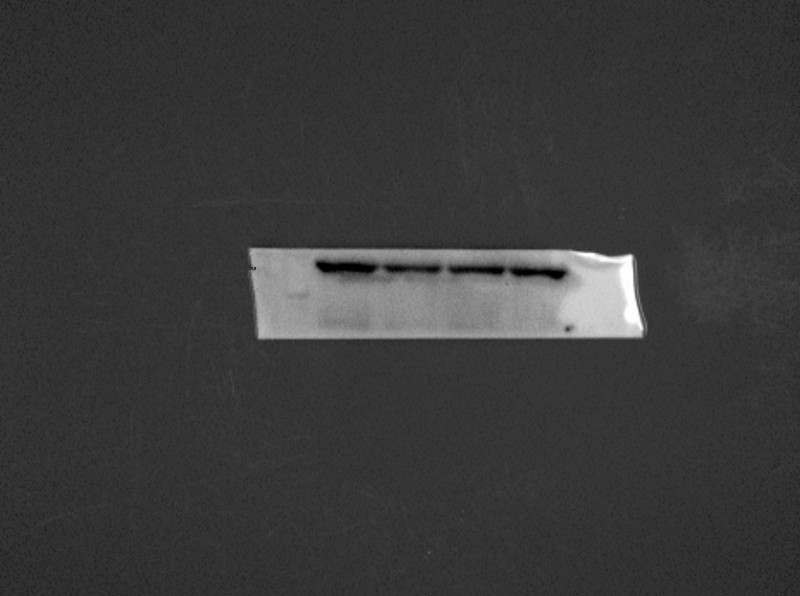

Supplement: Supplementary file 1 — Supplementary file1 (ZIP 2112 KB) [file 210_2023_2883_MOESM1_ESM.zip › full uncropped Gels and Blots image/prss35/1.jpg]

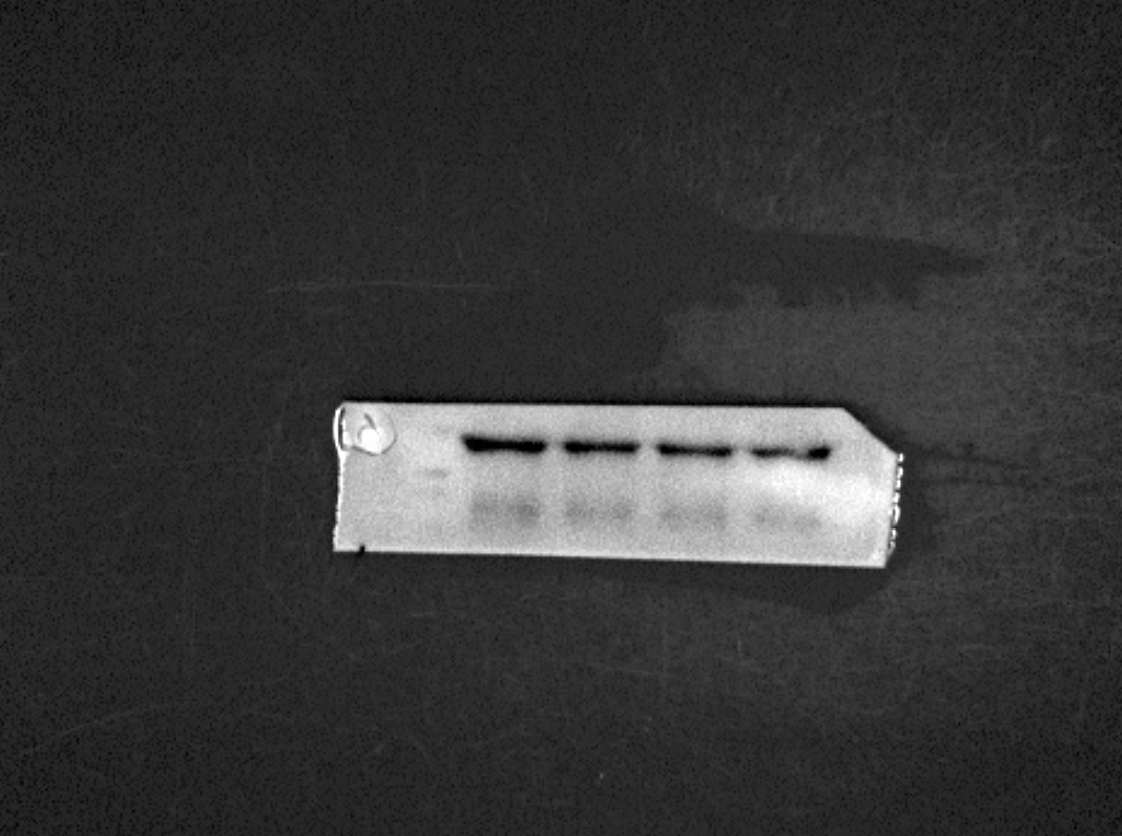

Supplement: Supplementary file 1 — Supplementary file1 (ZIP 2112 KB) [file 210_2023_2883_MOESM1_ESM.zip › full uncropped Gels and Blots image/prss35/2.jpg]

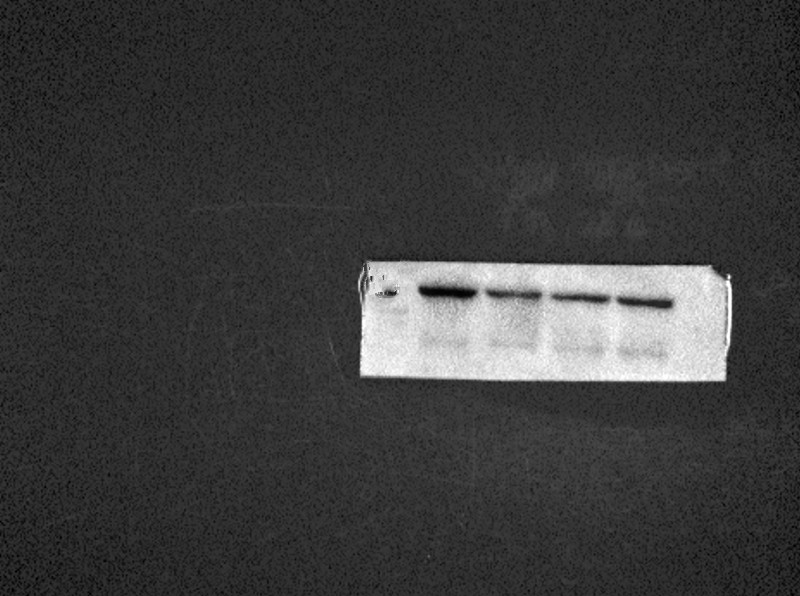

Supplement: Supplementary file 1 — Supplementary file1 (ZIP 2112 KB) [file 210_2023_2883_MOESM1_ESM.zip › full uncropped Gels and Blots image/prss35/3.jpg]

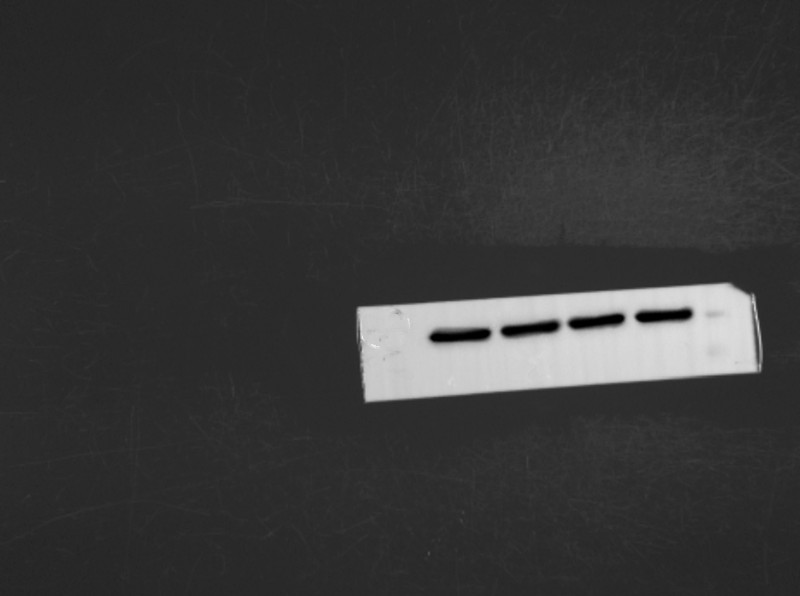

Supplement: Supplementary file 1 — Supplementary file1 (ZIP 2112 KB) [file 210_2023_2883_MOESM1_ESM.zip › full uncropped Gels and Blots image/a┬-actin/1.jpg]

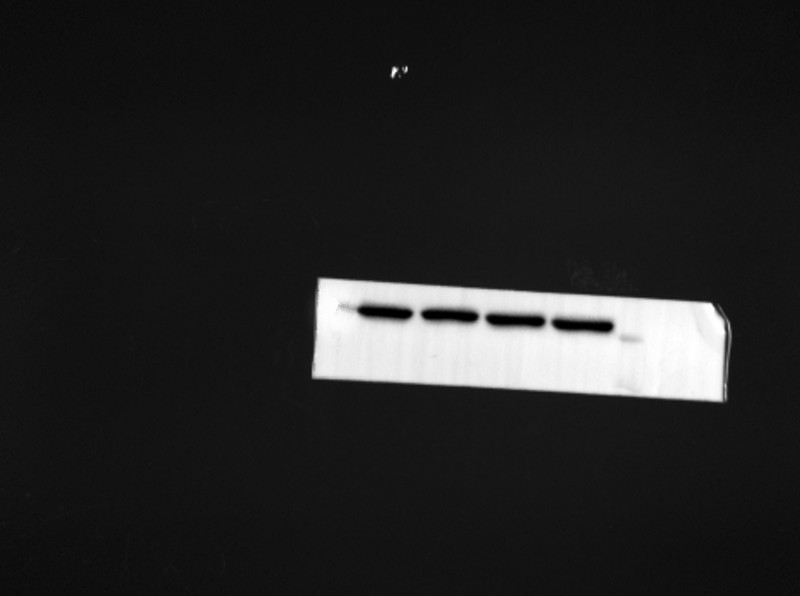

Supplement: Supplementary file 1 — Supplementary file1 (ZIP 2112 KB) [file 210_2023_2883_MOESM1_ESM.zip › full uncropped Gels and Blots image/a┬-actin/2.jpg]

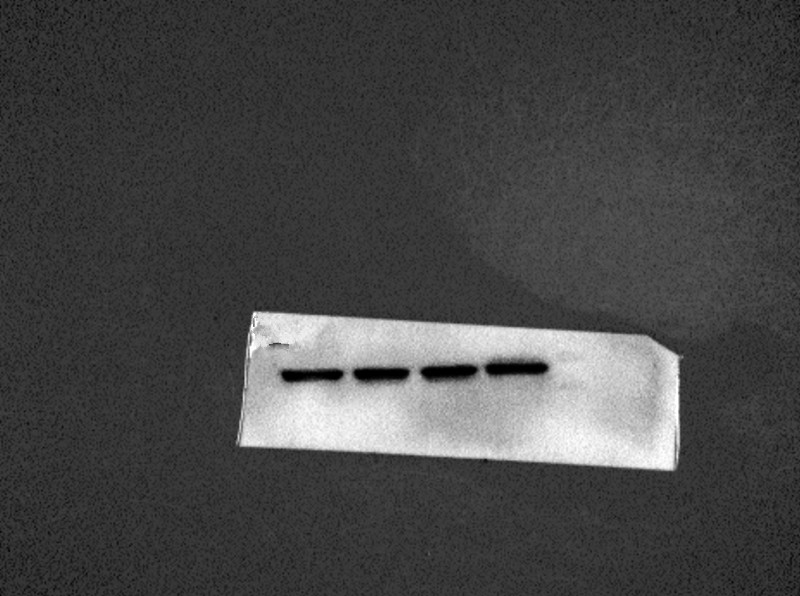

Supplement: Supplementary file 1 — Supplementary file1 (ZIP 2112 KB) [file 210_2023_2883_MOESM1_ESM.zip › full uncropped Gels and Blots image/a┬-actin/3.jpg]
